# Preliminary Study Using Wearable Near-Infrared Spectroscopy for Continuous Monitoring of Hemodynamics Through the Carotid Artery

**DOI:** 10.3390/bios15080549

**Published:** 2025-08-20

**Authors:** Nisha Maheshwari, Alessandro Marone, Lokesh Sharma, Stephen Kim, Albert Favate, Andreas H. Hielscher

**Affiliations:** 1 Department of Biomedical Engineering, 6 MetroTech Center, New York University Tandon School of Engineering, Brooklyn, NY 11201, USA; am11201@nyu.edu (A.M.); ls5940@nyu.edu (L.S.); hk3363@nyu.edu (S.K.); ahh4614@nyu.edu (A.H.H.); 2 CaroRhythm, Inc., 628 E 11th St, New York, NY 10009, USA; 3 Department of Vascular Neurology, New York University Grossman School of Medicine, 222 E 41st St Fl 10, New York, NY 10017, USA; albert.favate@nyulangone.org

**Keywords:** near-infrared spectroscopy, Biophotonics, tissue oxygen saturation, hemodynamics, carotid artery

## Abstract

Non-invasive, continuous monitoring of carotid artery hemodynamics may provide valuable insights on cerebral blood perfusion (CBP). Near-infrared spectroscopy (NIRS) is a non-invasive modality that may be a good candidate for real-time carotid artery monitoring. We designed a wearable NIRS system to monitor the left and right radial and carotid arteries in 20 healthy subjects. The changes in total hemoglobin concentration (HbT) and tissue oxygen saturation (StO_2_) in all 80 arteries were continuously monitored in response to changes in oxygen supply. Wilcoxon non-parametric equivalence testing was used to compare changes in the radial (reference) and carotid arteries. The system-derived HbT and StO_2_ trends matched the expected physiological responses over time in the radial and carotid arteries. The mean peak-to-peak amplitude [uM] of HbT during sustained deep breathing was practically equivalent between the left radial (0.9 ± 0.8) and left carotid (1.6 ± 1.1) arteries (*p* = 0.01). The mean peak-to-peak amplitude [%] of StO_2_ was practically equivalent between the left radial (0.3 ± 0.2) and left carotid (0.3 ± 0.2) arteries (*p* < 0.001) and the right radial (0.4 ± 0.5) and right carotid (0.5 ± 0.4) arteries (*p* = 0.001). These findings indicate that NIRS may be a good option for monitoring the carotid arteries to track changes in CBP.

## 1. Introduction

The carotid arteries are critical for supplying blood to the brain and have been identified as good candidates for measuring cerebral blood perfusion (CBP) [1]. These arteries respond immediately to local changes in CBP via the carotid body, a sensory organ that detects changes in blood oxygen saturation (SpO_2_) through the artery [1,2]. Monitoring the carotid arteries could therefore help identify irregularities in blood flow and SpO_2_ that may be indicative of cardiovascular diseases such as large vessel occlusions (LVOs), which are most commonly related to carotid artery stenosis [3,4,5,6].

Research efforts are underway to identify the best techniques for imaging and monitoring of the carotid arteries. Huang et al. (2023) developed a novel image segmentation technique to measure intima-media thickness of the carotid artery, a metric closely tied to cardiovascular disease [7]. Their nested attention-guided deep learning model achieved high accuracy in identifying key carotid artery features. However, their model uses existing ultrasound images taken by an experienced clinician in-hospital and has not been tested for dynamic or real-time capabilities. Wang et al. (2021) developed an impedance plethysmography (IPG)-based carotid pulse sensing device for continuous blood pressure monitoring [8]. The study sample size was only six subjects, and the results were not compared to gold standard blood pressure measurements, making it difficult to ascertain the accuracy of the device. A wearable ultrasound probe capable of multi-day monitoring was designed and tested on the carotid artery by Frey et al. (2022) [9]. Their probe was low-power, wireless, and compact. However, similar to in-hospital ultrasound systems, this device had to be held in place by the study team during testing and was not flexible, which significantly impacts wearability. Despite the ongoing research in the field, there remains a gap in non-invasive monitoring techniques that provide accurate, real-time information on hemodynamic changes in the carotid arteries.

Near-infrared spectroscopy (NIRS) may provide a low-power, inexpensive option for real-time, dynamic carotid artery monitoring that could be applied to an outpatient setting. We have previously demonstrated the use of NIRS for monitoring blood flow through arteries as deep as 7 mm below the surface of the skin in patients with peripheral arterial disease [10,11,12,13]. The carotid arteries are much deeper below the skin’s surface (>20 mm) [14,15,16], which was previously thought to be a barrier for using NIRS to monitor these arteries. In this paper, we present a preliminary study with NIRS-derived hemodynamic data from the left and right radial and carotid arteries of 20 healthy subjects. Data were acquired continuously and simultaneously from all four locations during different structured breathing exercises. We determined the equivalence in changes over time in blood flow and tissue oxygen saturation (StO_2_) between the radial and carotid arteries, with the radial arteries serving as the references for comparison. The primary objective of this study was to demonstrate that our NIRS system can monitor changes in blood perfusion and StO_2_ related to the carotid arteries.

## 2. Materials and Methods

### 2.1. Study Cohort Population

For this study, we enrolled 20 subjects, as previous studies utilizing our NIRS system showed promising results in diagnosing and monitoring vascular diseases in cohorts of 14 and 20 patients [10,11]. Each subject was scheduled for a single visit that took place between 2023 and 2024 at the New York University (NYU) Langone Tisch Hospital in New York, NY, USA.

Enrolled subjects were healthy individuals between the ages of 18–40 who were recruited via postings on iConnect (https://clinicaltrials.med.nyu.edu/ (accessed on 17 August 2025)) and ResearchMatch (https://www.researchmatch.org/ (accessed on 17 August 2025)), the two NYU Grossman School of Medicine IRB-approved sites that match volunteers with research studies. Having subjects independently express interest in the study helped to minimize selection bias. Subjects were excluded from the study if they had a history of vascular disease, smoking, neck trauma, neck radiation treatment, hypertension, diabetes, or high cholesterol. Healthy subjects who do not have risk factors for cardiovascular disease and do not have carotid stenosis are expected to have similar changes in hemodynamics between the radial and carotid arteries in response to external stimuli. Thus, the radial arteries were used as reference arteries for this study. Of the 20 subjects in the study cohort, 65% of subjects were assigned female at birth and 70% of subjects were non-white (Table 1), which mirrors differences in stroke prevalence (a common result of LVOs) across sex and race [17,18,19]. We distinguished between race and skin tone, and classified each subject’s complexion as fair, olive, or dark. In an attempt to address the subjectivity in assigning skin tone designators, the same study team member categorized skin tones for all subjects.

### 2.2. NIRS System

**Figure 1:**
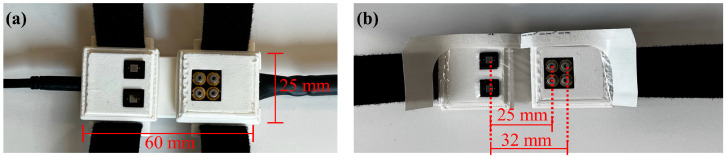
(**a**) Patch that sits over the left and right radial arteries. (**b**) Patch that sits over the left and right carotid arteries. Both patches are 60 mm × 25 mm in size and have the same source-detector distances of 25 mm and 32 mm.

The NIRS system used in this study has four sensing modules, or “patches”. Each patch is approximately 60 mm × 25 mm in size and has four laser diodes configured in a 2 × 2 square. The diodes emit light at wavelengths of 670 nm, 780 nm, 808 nm, and 850 nm (L780P010, L808P010, HL6748MG, L850P010, Thorlabs). These wavelengths were selected based on the known absorption spectra of oxyhemoglobin (HbO_2_) and deoxyhemoglobin (Hb), which are well differentiated from other chromophores in the 650 nm–900 nm range. The light that diffuses in the tissue is measured by two silicon photodiodes (S1337-33BR, Hamamatsu) located at distances of 25 mm and 32 mm from each column of laser diodes, respectively. This allows for a penetration depth of up to 16 mm, which is sufficient to monitor the tissue above the carotid bulb in most cases [14,15,16]. The patches were placed on the skin above the left and right radial arteries (Figure 1a) and the left and right carotid arteries (Figure 1b).

The NIRS system is controlled by a laptop with a custom-designed MATLAB graphical user interface capable of continuously displaying the total hemoglobin (HbT) measured by all of the patches. Data from all four patches are collected simultaneously at a rate of 2.24 Hz. A diffusion-theory based algorithm uses the patch signals as inputs to reconstruct the HbO_2_, Hb, and HbT concentrations in real-time and extract clinically relevant parameters, such as the StO_2_.

### 2.3. Study Design and Data Collection

**Figure 2:**
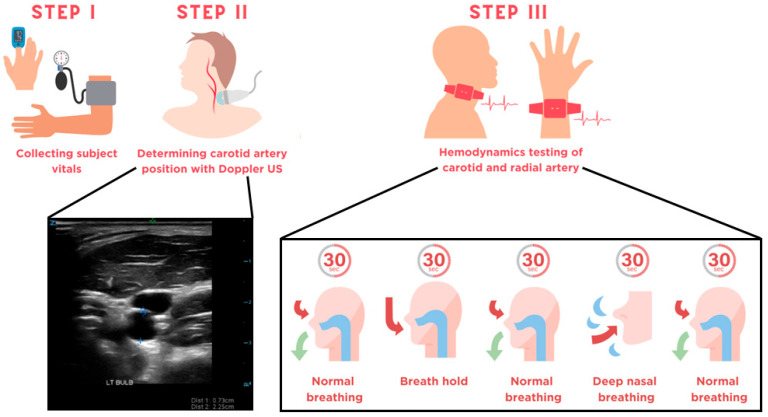
Infographic detailing the data acquisition protocol. In Step I, the heart rate, blood pressure, and oxygen saturation are taken in the supine position. In Step II, transverse images of the carotid bulb, internal carotid artery, and external carotid artery on the left and right sides of the subject are taken. Finally, in Step III the subject was instructed to perform a series of breathing exercises, during which their left and right radial and carotid arteries were monitored with the NIRS system.

Data collection for the study consisted of three steps (Figure 2). In Step I, vital signs including heart rate, systolic and diastolic blood pressure, and blood oxygen saturation were obtained from subjects at rest in the supine position. The heart rate [beats per minute] and blood pressures [mmHg] were found using an automatic blood pressure sensor (BP5450, Omron Healthcare, Hoffman Estates, IL, USA), while a fingertip pulse oximeter (500BL, Zacurate, Stafford, TX, USA) was used to validate the heart rate and obtain the subject’s blood oxygen saturation.

In Step II, an ultrasound technician identified the locations of the left and right carotid arteries (Figure 2) and recorded the depth and diameter of the carotid bulbs and the diameters of the internal carotid arteries (ICAs) and external carotid arteries (ECAs) shown in Supplemental Table S1. A small mark was made on the skin at the bifurcation points of the left and right carotid arteries using a surgical marker.

The four patches were placed on the tissues above the left and right carotid and radial arteries at the start of Step III (Figure 2). The bifurcation mark was used to center the patches on the carotid arteries and ensure that they were positioned on the tissue directly above the carotid bulb. The carotid arteries are the primary suppliers of blood to the neck, and this placement in addition to the penetration depth of 16 mm ensured that measured changes in HbT and StO_2_ by the NIRS system were reflective of changes in the carotid arteries [20,21]. The radial arteries on the left and right sides were visually identified in each subject and the patches were placed accordingly. The same patch was used for the same location across all subjects to account for the effects of system error on the acquired data. Patches were adhered to the tissue of the subject via resizable bands and/or medical tape (3M Tegaderm Film 1626W, Solventum, St. Paul, MN, USA). All measurements acquired with the NIRS system were recorded with the subject in the supine position.

For each trial, subjects were asked to perform a series of breathing exercises while wearing the patches. The breathing sequence was as follows: (1) 30 s of normal, controlled breathing; (2) ~5 s inhalation followed by a ~25 s breath hold; (3) release and 30 s of normal, controlled breathing; (4) 30 s of deep breathing through the nose (at the subject’s regular/chosen inhale-exhale frequency); and (5) 30 s of normal, controlled breathing. The total time for one trial was 2.5 min, during which the subjects were instructed not to move or speak and to minimize swallowing. The trials were performed 3–4 times with each subject, and the patches remained in the same position on a subject for all trials.

We identified six parameters of interest to compare the changes in HbT and StO_2_ between the carotid and radial arteries in response to the breath hold (BH) and deep breathing (DB) exercises (Table 2).

**Figure 3:**
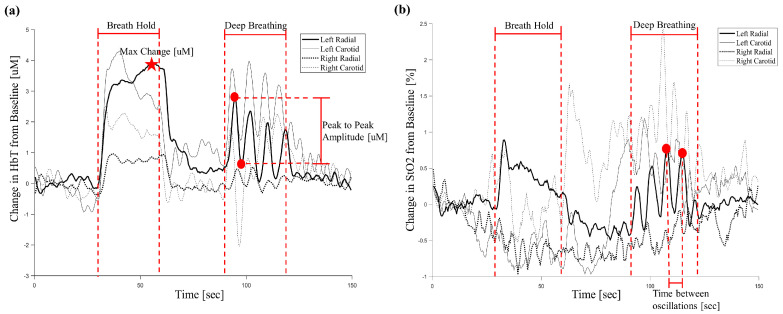
(**a**) The change in HbT [uM] from baseline over time for a representative trial. The breath hold (BH) and deep breathing (DB) segments of the trial are indicated. The red star marks the maximum change in HbT [uM] during the BH. The red dots mark a characteristic peak-to-peak amplitude [uM] of one oscillation. (**b**) The change in StO_2_ [%] from baseline over time for a representative trial. The BH and DB segments are indicated. The red dots mark a characteristic time between oscillations [sec]. For clarity, the left radial artery was used as an example to highlight parameters of interest, and all parameters were calculated for both features (HbT and StO_2_) at all locations.

The maximum % change parameters compare the maximum values of HbT and StO_2_ during the BH with the mean “baseline” values of HbT and StO_2_ during the first 20 s of normal breathing (Figure 3). The parameters corresponding with the DB segment of the trial compare the oscillatory patterns (which are related to the cycles of inhalation and exhalation) of the HbT and StO_2_ curves at each location (Figure 3). The mean oscillation time is defined as the mean time between one peak/trough to the next during DB and the mean peak-to-peak amplitude is defined as the difference in values (either uM or % for HbT and StO_2_, respectively) between a peak and corresponding trough during DB.

### 2.4. Statistical Analysis

Multiple trials were conducted per subject to ensure that at least one trial had minimal noise due to movement or machine error. Analyses for this paper were performed on the “least noisy” trial data from each subject. These data were determined by calculating the signal-to-noise ratio (SNR) of the measured HbT data during the DB segment of each trial. MATLAB’s (MATLAB 2023; The MathWorks, Inc., Natick, MA, USA) existing “snr” function was used to compute the SNR; the function assumes the measurement trace is sinusoidal and has a known sampling rate, both of which are true during the DB segment of the trial. In the cases where SNR was less than 1 for all the trials at a specific location, the subject’s data at that location was not used for analysis. Outliers from the six parameters of interest were discarded during analysis if they were not within two standard deviations of the mean. Parameters at each location are reported as mean ± standard deviation.

All test statistics were adjusted according to the Bonferroni correction protocol. The reported *p*-values were adjusted based on the number of tests per location for each parameter. Data were considered statistically significantly equivalent when P_adjusted_ ≤ 0.05. RStudio (RStudio release 2024.09.1+394.pro7; RStudio, Boston, MA, USA) was used for equivalence testing between the left radial and left carotid arteries, the right radial and right carotid arteries, the left and right radial arteries, and the left and right carotid arteries. The distributions of the parameters of interest were nonnormal and RStudio’s TOSTER package was used to run the Wilcoxon-specific continuity corrected two one-sided tests (Wilcox-TOST) analysis. Thresholds for practical equivalency were determined based on the standard deviations of the datasets of interest. The median of differences (MoD) and 95% confidence interval (CI) are reported with the P_adjusted_-values as MoD [95% CI]. RStudio’s PowerTOST package was used for the sample size calculation.

Kendall rank correlation analysis was performed in RStudio to determine the linear dependencies between the NIRS system-derived parameters of interest and the heart rate, systolic blood pressure, diastolic blood pressure, and carotid artery measurements from each subject (Table 1, Table S1). Two parameters were considered marginally correlated if the correlation coefficient |τ| > 0.2 and moderately correlated if the correlation coefficient |τ| > 0.4. Only correlation coefficients with statistical significance (P_adjusted_ ≤ 0.05) are reported, unless otherwise stated. RStudio was also used to perform the Kruskal-Wallis test to determine the effect of categorical factors such as the sex assigned at birth, race, and skin tone of the subject population on the system-derived parameters. These demographic factors were determined to have an effect on a parameter if there was a statistically significant difference (P_adjusted_ ≤ 0.05) in parameter values between different demographic categories, and the effect size was found using Kruskal’s η^2^ calculation. Only three categorizations were used for skin tone due to the size of the patient cohort. The sample size was too small to use a model such as the Fitzpatrick scale and perform statistical analysis with (N > 1) in each category.

## 3. Results

### 3.1. Trends in HbT Concentration and StO_2_

Changes in HbT and StO_2_ followed similar patterns across the left and right radial and carotid arteries, and these changes matched what were physiologically expected in response to the breathing changes in most subjects (Figure 3). There was a characteristic increase in HbT during the BH at all locations to maintain the oxygen demands of the tissue (Figure 3a). The StO_2_ did not consistently increase or decrease during the BH (Figure 3b).

Both the HbT and the StO_2_ oscillated during the DB at all locations in response to the inhalation and exhalation of the subject. The frequency of StO_2_ oscillations matched the inhalation and exhalation cycles of the 20 subjects, and the HbT oscillations demonstrated an expected characteristic delay as they were responding to changes in StO_2_. Both the HbT and StO_2_ appeared to return to their initial baseline values during the final normal breathing segment immediately following the DB.

There were no statistically significant correlations between any of the six system-derived parameters (Table 2) and demographic information including the heart rate, systolic blood pressure, diastolic blood pressure, sex assigned at birth, race, and skin tone (Table 1). We further confirmed that the effect sizes of these demographic factors on all six parameters were negligible, with η^2^ < 0.05. Analysis was also performed to determine statistically significant correlations between NIRS-derived parameters from the carotid arteries and the anatomical features of those arteries (Table S1). The maximum % change in HbT at the left carotid artery during the BH was moderately correlated with the diameters of the left ICA (τ = 0.50) and the left ECA (τ = 0.42).

### 3.2. Comparison of NIRS System-Derived Parameters Across Locations

Wilcox-TOST analysis was used to determine the equivalence between the reference arteries (the radial arteries) and the arteries of interest (the carotid arteries) for each of the parameters identified.

**Figure 4:**
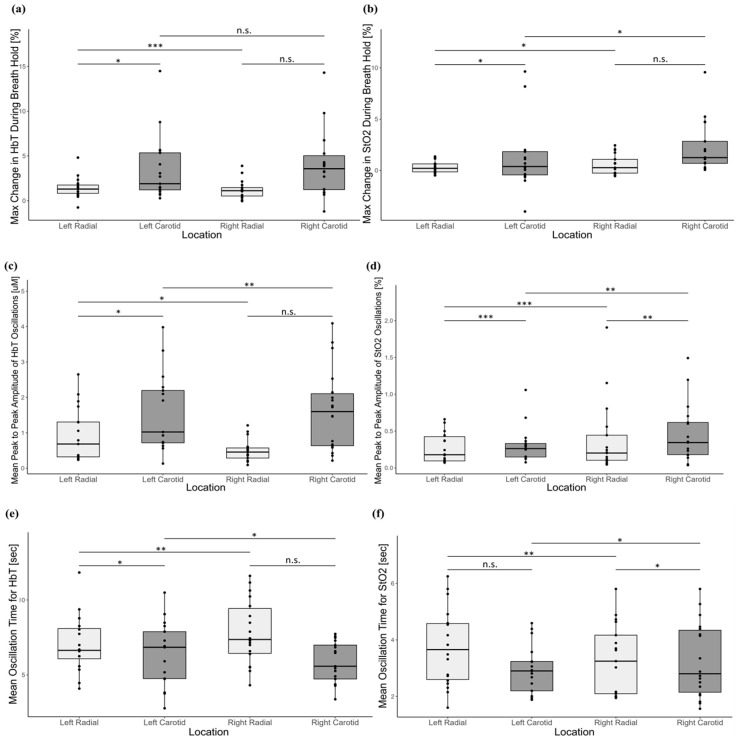
The maximum percentage change in (**a**) HbT [%] and (**b**) StO_2_ [%], the mean peak-to-peak amplitude of (**c**) HbT [uM] and (**d**) StO_2_ [%], and the mean oscillation time for (**e**) HbT [sec] and (**f**) StO_2_ [sec]. The following arteries are compared for equivalency: (1) the left radial and left carotid; (2) the right radial and right carotid; (3) the left and right radials; and (4) the left and right carotids. Arteries were compared using the Wilcox-TOST equivalence test. n.s. = no significant equivalence; *= *p* ≤ 0.05; **= *p* < 0.01; ***= *p* < 0.001.

The left radial and left carotid arteries showed statistically significant equivalence during the BH and DB segments for the changes in both HbT and StO_2_. The maximum % changes in HbT during BH (Figure 4a) were statistically significantly equivalent between the left radial (1.4 ± 1.1%) and the left carotid (3.5 ± 3.7%) arteries (MoD, −0.94 [−3.87, 0.23]; *p* = 0.05) with a threshold of 3.7%. Similarly, the maximum % change in StO_2_ during BH (Figure 4b) was statistically significantly equivalent between the left radial (0.3 ± 0.6%) and left carotid (1.2 ± 3.2%) arteries (MoD, −0.26 [−2.80, 0.81]; *p* = 0.03) with a threshold of 3.2%.

The mean peak-to-peak amplitudes for HbT (Figure 4c) between the left radial (0.9 ± 0.8 uM) and left carotid (1.6 ± 1.1 uM) arteries were statistically significantly equivalent (median of differences (MoD), −0.41 [−0.96, 0.08]; *p* = 0.01) with a threshold of 1.2 uM. Similarly, the mean peak-to-peak amplitudes for StO_2_ (Figure 4d) between the left radial (0.3 ± 0.2%) and left carotid (0.3 ± 0.2%) arteries were statistically significantly equivalent (MoD, −0.002 [−0.004, 0.002]; *p* = 0.0007) with a threshold of 0.5%. The mean oscillation times of HbT (Figure 4e) between the left radial (7.0 ± 1.9 s) and left carotid (6.5 ± 2.1 s) arteries were statistically significantly equivalent (MoD, 0.34 [−0.82, 1.61]; *p* = 0.02) with a threshold of 2.1 s. The mean oscillation time of StO2 (Figure 4f) was the only parameter that was not practically equivalent between the left radial (3.7 ± 1.4 s) and left carotid (3.0 ± 0.8 s) arteries (MoD, 0.79 [−0.34, 1.88]; *p* = 0.3) with a threshold of 1.4 s.

The right radial and right carotid arteries showed statistically significant equivalence only for changes in StO_2_ during DB. The mean peak-to-peak amplitudes for StO_2_ (Figure 4d) between the right radial (0.4 ± 0.5%) and right carotid (0.5 ± 0.4%) arteries were statistically significantly equivalent (MoD, −0.0005 [−0.002, 0.001]; *p* = 0.001) for a threshold of 0.5%. The mean oscillation times of StO2 (Figure 4f) were also statistically significantly equivalent (MoD, −0.29 [− 1.31, 1.02]; *p* = 0.02) between the right radial (3.3 ± 1.2 s) and right carotid (3.3 ± 1.3 s) arteries with a threshold of 1.4 s. The means, standard deviations, and the equivalence testing for the BH segment and the difference in changes in HbT during DB between the right radial and carotid arteries can be found in Supplemental Tables S2 and S3, respectively. Tables S2 and S3 also include the means, standard deviations, and equivalence testing between the left and right radial arteries and between the left and right carotid arteries for all parameters.

## 4. Discussion

### 4.1. Trends in HbT Concentration and StO_2_ in Response to Breathing Exercises

The HbT concentration [uM] and StO_2_ [%] curves that were reconstructed from the data acquired by the NIRS system displayed the physiologically expected responses to different breathing exercises (Figure 3). During a BH, the buildup of carbon dioxide and the decrease in blood oxygen saturation affect the chemoreceptors in the aortic arch and carotid bodies, which can facilitate vasodilation—resulting in an increase in HbT—to maintain the necessary oxygen supply to the brain and extremities [22,23,24,25]. We observed this trend in HbT in both the left and right radial and carotid arteries consistently across subjects during the BH [see characteristic curve in Figure 3a]. Changes in StO_2_ did not follow a consistent trend across subjects during the BH. The magnitude of the change in StO_2_ was less than that of HbT for all locations. There was a marginal correlation between maximum% change in HbT during BH and maximum% change in StO_2_ during BH (τ = 0.20), implying that a greater increase in HbT corresponded to an increase in StO_2_ in subjects. Due to the length of the BH segment, it is likely that the increase in HbT partially compensated for any decreases in StO_2_ that may have occurred due to restricted oxygen supply.

Both the HbT concentration [uM] and StO_2_ [%] curves exhibited the expected physiological behaviors during the DB segment as well (Figure 3). There were clear oscillations in response to deep inhalations and exhalations by the subjects. DB has been linked to increased efficiency in venous return, which is likely to have contributed to the observed increases in HbT and decreases in StO_2_ (due to increases in Hb concentration) [26,27]. The oscillation frequency during the DB for all locations was around twice as high in StO_2_ (Figure 4f) than HbT (Figure 4e). As with the BH, chemoreceptors in the aortic arch and carotid bodies are particularly susceptible to changes in StO_2_ and will facilitate changes in HbT in response [22,23,24,25]. Although chemoreceptors activate almost instantaneously to changes in StO_2_, the changes in HbT can take seconds [28,29,30]. This would explain the difference in oscillation frequency between HbT and StO_2_. The StO_2_ follows the breathing patterns of the subject and the HbT responds, with delay, to these changes in StO_2_.

### 4.2. Demographic and Anatomical Factors

Of the 20 healthy subjects in this study, 65% were assigned female at birth, 70% were nonwhite, 40% had a fair complexion, 40% had an olive complexion, and 20% had a dark complexion. There were no correlations between system-derived parameters and the sex assigned at birth, the race, or the skin tone of the subject. The effect sizes of these factors were negligible, with η^2^ < 0.05 for all factors. These preliminary results may suggest that our system is robust enough to acquire data from diverse subject populations.

The left ICA and left ECA were the only anatomical factors moderately correlated with system-derived parameters, and only with maximum% change in HbT during BH (τ = 0.50 and τ = 0.42, respectively). These results suggest that our NIRS system is able to track changes in HbT and StO_2_ in the tissue surrounding the carotid arteries irrespective of depth and size of the arteries.

### 4.3. Equivalence Testing of System-Derived Parameters

Large standard deviations were observed in the two BH parameters (Figure 4a,b) and in the mean peak-to-peak amplitudes of HbT and StO_2_ during DB (Figure 4c,d). This may be due to the effects of subject-specific physiology: lung capacity, tissue oxygen demand, and arterial shape all differ at an individual level. These factors affect the blood flow and oxygen saturation throughout the arteries [4,31,32], which may explain the large standard deviations observed in the system-derived parameters. In comparison, the mean oscillation times for both HbT and StO_2_ during DB had much smaller standard deviations relative to the mean. It is likely that there is less variation in the length of a typical deep inhalation and exhalation between individuals, making the mean oscillation times for StO_2_ and HbT more standardized than the magnitude of changes in HbT or StO_2_ in healthy subjects.

The oxygen demands of the tissues in the brain are greater than those of the tissues in the peripheral extremities, so we did not expect the absolute concentrations of HbT to be the same in the carotid and radial arteries. The carotid and radial arteries on the left side of the body originate from the aortic arch and the carotid and radial arteries on the right side of the body originate from the brachiocephalic artery, which follows immediately after the aortic arch. In the absence of factors such as carotid stenosis, which could cause local changes in blood flow, we expected changes in HbT and StO_2_ to be practically equivalent between the radial and carotid arteries on either side of the body. However, this anatomical difference may account for the lack of equivalence of the maximum% change in HbT between the left and right carotid arteries (MoD, −0.95 [−3.96, 2.04]; *p* = 0.07).

During testing, we faced an issue with patch adherence to the right carotid artery. The necks of some subjects were too small for our resizable bands to accommodate. In these cases, the band that was connected to the right carotid artery patch was held taut via Tegaderm, and the Tegaderm was always placed over the patch on the left carotid artery. As a result, the patch on the left carotid artery had better adhesion, which may explain the lack of equivalence between the right radial and right carotid arteries in all but two parameters—the mean peak-to-peak amplitude of StO_2_ (*p* = 0.001) and the mean oscillation time of StO_2_ (*p* = 0.02). Additionally, the right radial artery can experience more tortuosity in blood flow relative to the other arteries of interest due to anatomical variation [33,34,35], which may further contribute to the differences between the right radial and right carotid arteries.

The left radial and left carotid arteries had practical equivalence during both the BH and DB exercises. The maximum% change in HbT, the maximum% change in StO_2_, the mean peak-to-peak amplitude of HbT, the mean peak-to-peak amplitude of StO_2_, and the mean oscillation time for HbT were all statistically significantly equivalent between these two arteries (*p* = 0.05, *p* = 0.03, *p* = 0.01, *p* = 0.0007, and *p* = 0.02, respectively). The left radial and left carotid arteries lacked statistically significant equivalence only for the mean oscillation time for StO_2_ [*p* = 0.3; Figure 4f]. The differences in mean oscillation time of StO_2_ between the two arteries was larger than the threshold used for the Wilcox-TOST statistical analysis, which may account for the lack of equivalence.

### 4.4. Study Limitations

Subject movement during the testing was a major study limitation. The NIRS system designed by our lab was a low-fidelity prototype and the measurement patches were attached to the control board and computer via wires. Patch measurements were susceptible to movements in the wires, which were laid across the chest of the subject during testing. Although subjects were instructed not to move or speak during the testing, movements from swallowing and gulping were largely unavoidable. These actions caused the wires at all locations to move and affected the adhesion of the carotid patches to the skin in some cases. Poor adhesion was another limitation, as the resizable straps used in this study were not sufficient for all cases. The patch on the right carotid artery was particularly impacted, as the adhesion was consistently better for the left carotid artery patch (see Section 4.3). Outliers in the data may have been due to movement or adhesion problems rather than physiological differences.

It has been shown that BH and DB exercises affect heart rate, systolic blood pressure, and diastolic blood pressure, and these factors affect blood flow [23,24,36]. However, we did not continuously monitor heart rate, systolic blood pressure, or diastolic blood pressure during the breathing exercises. In future studies, we will monitor these three parameters continuously to confirm the physiological responses to the breathing exercises and more strongly validate the changes observed by our NIRS system.

## 5. Conclusions

The data derived from our NIRS-system measurements matched the expected physiological changes in the left and right carotid arteries in response to BH and DB exercises. Additionally, the trends for HbT and StO_2_ differed from each other in the expected ways. The NIRS-derived parameters maximum % change in StO_2_ during BH and mean peak-to-peak amplitude of StO_2_ during DB both directly reflect the changes in oxyhemoglobin delivery to the brain via the carotid arteries over time. This is critical given that CBP is responsible for delivering arterial blood to the brain [37]. The NIRS-derived parameters maximum % change in HbT during BH and mean peak-to-peak amplitude of HbT during DB allow for the extrapolation of CBP via concentration-versus-time curves [38]. The strong correlation between our NIRS-derived parameters and known physiological changes indicates that our NIRS system may be capable of monitoring both oxygen saturation and blood flow, two important and distinct metrics that can better inform CBP and cardiovascular risk.

The left radial and carotid arteries were statistically significantly equivalent across five of the parameters of interest, and the right radial and carotid arteries were statistically significantly equivalent across the two StO_2_ parameters from the DB segment. Based on the results observed in this study, a larger sample size is needed to reject the null equivalence hypothesis for all comparisons and determine if the lack of equivalency across the other parameters was due to poor adhesion, noise, or the anatomy of the right radial artery. A sample size of 54 subjects would be necessary to show statistically significant equivalence (with the thresholds used in this paper) for all four artery pairs (left radial and carotid, right radial and carotid, left and right radial, and left and right carotid) across all six parameters with a power of 0.8 at α = 0.05 [39].

Given the practical equivalence between the left radial and carotid arteries, the left and right radial arteries, and the left and right carotid arteries (Table S3) for the identified parameters of interest, these preliminary results inspire confidence that our system can track changes through the carotid arteries in real time. We will focus our technical development on the design of a wireless and better adhering NIRS probe. Power consumption and weight will need to be considered when transitioning to a wireless system. These considerations may be addressed by switching from laser diodes to low-power LEDs with similar radiant power profiles. Additionally, biocompatibility of the electronic housing and adhesive will ensure that long-term wear is possible without adverse effects on the subject. Future prototypes that are wireless and better adhere to the neck may be good candidates for non-invasive, real-time monitoring of CBP and hemodynamics through the carotid arteries.

## 6. Patents

PCT patent application no. PCT/US2024/038690 is related to this work.

## Figures and Tables

**Table 1:** Study Population Demographics, Given as the Number of Subjects and Percentage of Total Subjects or as the Mean ± Standard Deviation (std.) for the Study Cohort (N = 20).

Characteristics	Total
Sex assigned at birth—female	13 (65%)
Race—nonwhite	14 (70%)
Systolic blood pressure—mmHg	115 ± 11
Diastolic blood pressure—mmHg	71 ± 7
Heart rate—beats per minute	72 ± 11

**Table 2:** Parameters of Interest and their Corresponding Breathing Exercise.

Breathing Segment	Parameter
Breath Hold (BH)	Maximum % change [%] in feature ^1^
Deep Breathing (DB)	Mean oscillation time [sec] for feature
	Mean peak-to-peak amplitude [uM or %] of feature

^1^ Each parameter is associated with two features: total hemoglobin (HbT) and tissue oxygen saturation (StO_2_) for a total of six parameters of interest.

## Data Availability

The data presented in this paper can be made available for non-commercial use upon reasonable request, after acceptable completion of the NYU data use agreement. Requests to access the data should be directed to the corresponding author.

## Supplementary Materials

The following supporting information can be downloaded at: https://www.mdpi.com/article/10.3390/bios15080549/s1, Table S1: carotid ultrasound measurements; Table S2: measurements for all parameters of interest; Table S3: Wilcox-TOST equivalence testing.

## Abbreviations

The following abbreviations are used in this manuscript:

BH	Breath hold
CBP	Cerebral blood perfusion
CI	Confidence interval
DB	Deep breathing
ECA	External carotid artery
Hb	Deoxyhemoglobin
HbO_2_	Oxyhemoglobin
HbT	Total hemoglobin
ICA	Internal carotid artery
LVO	Large vessel occlusion
MoD	Median of differences
NIRS	Near-infrared spectroscopy
NYU	New York University
SNR	Signal-to-noise ratio
StO_2_	Tissue oxygen saturation
Wilcox-TOST	Wilcoxon-specific continuity corrected two one-sided tests
