# Supplementary material for: Preliminary Study Using Wearable Near-Infrared Spectroscopy for Continuous Monitoring of Hemodynamics Through the Carotid Artery
Source: Biosensors (Basel). 2025 Aug 20;15(8):549. doi: 10.3390/bios15080549 (PMC12384115; doi:10.3390/bios15080549)
Supplement: Supplementary file 1 [file biosensors-15-00549-s001.zip › biosensors-3810593-supplementary.pdf]

Supplemental Materials

Tables S1-S3

Table S1. Carotid artery measurements from doppler ultrasound in cm. Data are given as the mean ± standard deviation (std) for the study cohort (N = 20).

Anatomical Feature	***Location***	***Mean ± std. [cm]***
Bulb Depth	Left	1.51 ± 0.32
	Right	1.45 ± 0.32
Bulb diameter	Left	0.83 ± 0.13
	Right	0.84 ± 0.14
ICA* diameter	Left	0.70 ± 0.11
	Right	0.71 ± 0.11
ECA† diameter	Left	0.50 ± 0.08
	Right	0.48 ± 0.10

*ICA = internal carotid artery; †ECA = external carotid artery

Table S2 Values (given as mean ± standard deviation) for each parameter of interest at each location.

Parameter [units]	*Side*	*Location*	*Value*
Max % change of HbT [%]	Left	Carotid	3.5 ± 3.7
		Radial	1.4 ± 1.1
	Right	Carotid	4.0 ± 3.7
		Radial	1.2 ± 1.0
Max % change of StO2 [%]	Left	Carotid	1.2 ± 3.2
		Radial	0.3 ± 0.6
	Right	Carotid	2.2 ± 2.5
		Radial	0.6 ± 1.0
Mean peak-to-peak amplitude of HbT [uM]	Left	Carotid	1.6 ± 1.1
		Radial	0.9 ± 0.8
	Right	Carotid	1.7 ± 1.2
		Radial	0.5 ± 0.3
Mean peak-to-peak amplitude of StO2 [%]	Left	Carotid	0.3 ± 0.2
		Radial	0.3 ± 0.2
	Right	Carotid	0.5 ± 0.4
		Radial	0.4 ± 0.5
Mean oscillation time for HbT [sec]	Left	Carotid	6.5 ± 2.1
		Radial	7.0 ± 1.9
	Right	Carotid	5.8 ± 1.4
		Radial	7.8 ± 2.1
Mean oscillation time for StO2 [sec]	Left	Carotid	3.0 ± 0.8
		Radial	3.7 ± 1.4
	Right	Carotid	3.3 ± 1.3
		Radial	3.3 ± 1.2

Table S3 Wilcox-TOST equivalence testing for the following four artery pairs: left radial and carotid arteries, left and right radial arteries, left and right carotid arteries, and right radial and carotid arteries. Bolded p-values indicate locations that are statistically significantly equivalent.

Parameter [units]	δ^*	*Location 1*	*Location 2*	*MoD† [95% CI]*	*P-value*
Maximum % change in HbT‡ [%]	3.7	L radial	L carotid	−0.94 [−3.87, 0.23]	**0.05**
			R radial	0.43 [−0.37, 1.24]	**0.0007**
		L carotid	R carotid	−0.95 [−3.96, 2.04]	0.07
		R radial	R carotid	−2.71 [−5.27, −0.90]	0.44
Maximum % change in StO_2§ [%]	3.2	L radial	L carotid	−0.26 [−2.80, 0.81]	**0.03**
			R radial	−0.01 [−0.92, 0.67]	**0.0007**
		L carotid	R carotid	−1.41 [−2.67, 2.62]	**0.02**
		R radial	R carotid	−1.24 [−3.67, 0.24]	0.08
Mean peak-to-peak amplitude of HbT [uM]	1.2	L radial	L carotid	−0.41 [−0.96, 0.08]	**0.01**
			R radial	0.39 [0.01, 0.98]	**0.01**
		L carotid	R carotid	−0.20 [−0.74, 0.36]	**0.005**
		R radial	R carotid	−1.23 [−2.09, −0.51]	1
Mean peak-to-peak amplitude of StO_2 [%]	0.5	L radial	L carotid	−0.002 [−0.004, 0.002]	**0.0007**
			R radial	−0.0003 [−0.004, 0.002]	**0.0008**
		L carotid	R carotid	−0.0006 [−0.004, 0.0009]	**0.001**
		R radial	R carotid	−0.0005 [−0.002, 0.001]	**0.001**
Mean oscillation time for HbT [seconds]	2.1	L radial	L carotid	0.34 [−0.82, 1.61]	**0.02**
			R radial	−0.45 [−1.13, 0.13]	**0.002**
		L carotid	R carotid	0.87 [−0.28, 2.15]	**0.03**
		R radial	R carotid	1.80 [0.44, 3.15]	0.7
Mean oscillation time for StO_2 [seconds]	1.4	L radial	L carotid	0.79 [−0.34, 1.88]	0.3
			R radial	0.37 [−0.76, 1.47]	**0.05**
		L carotid	R carotid	−0.29 [−1.14, 0.62]	**0.02**
		R radial	R carotid	−0.29 [−1.31, 1.02]	**0.02**

*δ = Wilcox-TOST threshold; †MoD = median of differences; ‡HbT = total hemoglobin; §StO_2= tissue oxygen saturation
